# Stratification of systemic lupus erythematosus with IGHV4-34 in unswitched memory B cells

**DOI:** 10.1093/rheumatology/keaf386

**Published:** 2025-07-12

**Authors:** Toshiyuki Shiki Ushijima, Hiroyuki Teruya, Tatsuki Abe, Takahiro Itamiya, Mineto Ota, Tomohisa Okamura, Keishi Fujio

**Affiliations:** Department of Allergy and Rheumatology, Graduate School of Medicine, The University of Tokyo, Tokyo, Japan; Department of Allergy and Rheumatology, Graduate School of Medicine, The University of Tokyo, Tokyo, Japan; Department of Allergy and Rheumatology, Graduate School of Medicine, The University of Tokyo, Tokyo, Japan; Department of Functional Genomics and Immunological Diseases, Graduate School of Medicine, The University of Tokyo, Tokyo, Japan; Department of Functional Genomics and Immunological Diseases, Graduate School of Medicine, The University of Tokyo, Tokyo, Japan; Department of Functional Genomics and Immunological Diseases, Graduate School of Medicine, The University of Tokyo, Tokyo, Japan; Department of Allergy and Rheumatology, Graduate School of Medicine, The University of Tokyo, Tokyo, Japan

**Keywords:** B cells, systemic lupus erythematosus and autoimmunity, lymphocytes, biomarkers, immunosuppressants

## Abstract

**Objectives:**

SLE is an autoimmune disease characterized by the production of autoantibodies. Antibody affinities are defined by immunoglobulins, which include the immunoglobulin heavy-chain variable region (IGHV) genes, but the relationship between SLE and IGHV has not been fully elucidated. This study aimed to investigate the association between clinical features of SLE and IGHV.

**Methods:**

We recruited SLE patients and collected their peripheral blood. B cells from SLE patients were sorted into naïve B cells, unswitched memory (USM) B cells, switched memory B cells, double-negative B cells and plasmablasts. RNA-seq was performed to analyse IGHV gene usage, calculating the frequency of clonotypes that use IGHV4-34. The interactions between IGHV4-34 usage and clinical features were analysed by regression analysis, and the threshold for stratifying SLE patients by IGHV4-34 usage was determined by receiver operating characteristic (ROC) analysis.

**Results:**

IGHV4-34 usage in USM B cells had a significant association with disease activity. ROC curve analysis revealed the threshold of IGHV4-34 usage in USM B cells which distinguished patients with low complement levels from the other patients. High IGHV4-34 usage in USM B cells was associated with arthritis, haematuria, proteinuria, rash and fever. Achievement of a lupus low disease activity state (LLDAS), SLEDAI 2000 score and physician global assessment score showed worse values in the high IGHV4-34 usage group. Among those who achieved LLDAS under tacrolimus treatment, patients with high IGHV4-34 usage in USM B cells had a higher flare rate.

**Conclusion:**

IGHV4-34 usage in USM B cells is a potential biomarker of SLE and for patient stratification.

## Graphical abstract

**Figure:**
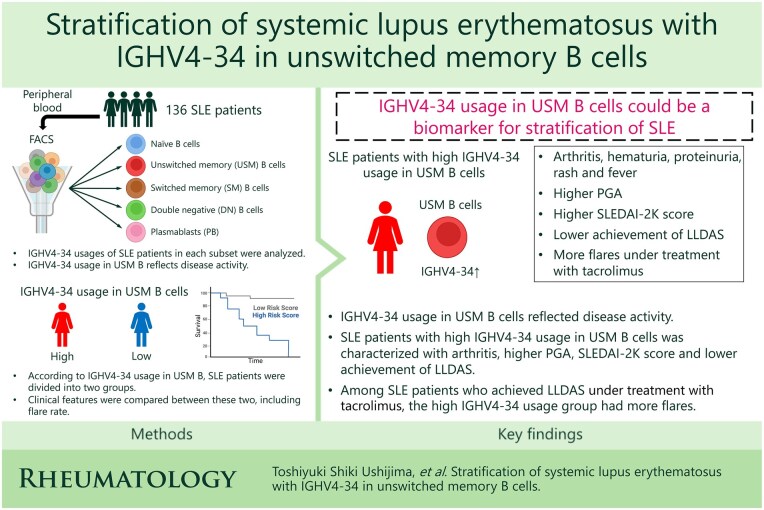

Rheumatology key messages

- IGHV4-34 usage in unswitched memory (USM) B cells can be used to stratify SLE patients.
- High IGHV4-34 usage in USM B cells was associated with severe disease activity.
- Among patients under tacrolimus treatment, the high IGHV4-34 usage group had more flares.

## Introduction

SLE is an autoimmune disease characterized by systemic inflammation. Physicians should treat patients according to the type of organ dysfunction complicating their SLE [1]. Some disease activity markers, such as anti-dsDNA antibody titres or decreased complement levels, have been identified, but they do not sufficiently reflect disease activity. As achieving low disease activity reduces the risk of organ damage in SLE [2], accurate evaluation of disease activity and proper patient stratification are needed.

Production of autoantibodies, such as anti-dsDNA and anti-Sm antibodies, is observed in SLE [3, 4]. Antibody affinity is determined by the B-cell receptor (BCR) sequence. BCR shows tremendous diversity due to genome editing, including VDJ recombination and somatic hypermutation (SHM) [5]. Normally, genome editing of BCR is strictly controlled, and B cells with self-reactive antibodies are eliminated by negative selection. This mechanism is defective in SLE [6].

Some autoreactive BCR sequences have been reported recently. Immunoglobulin heavy variable 4-34 (IGHV4-34) is one of the V genes of the heavy chain and is known to be involved in autoimmune diseases [7]. IGHV4-34 is a polyreactive BCR and is associated with protective responses against bacterial infection [8]. On the other hand, IGHV4-34 is also known to be associated with autoimmune diseases, including SLE, through the production of autoantibodies. In addition, IGHV4-34 usage in B cell is associated with disease activity of SLE patients [7, 9]. However, the association between specific SLE clinical features and IGHV4-34 is not well understood. Furthermore, B cells can be classified into several subsets, each of which play different roles, so the contribution of each subset to the disease should be discussed. In this study, we analysed IGHV4-34 in each B cell subset and aimed to clarify the associations between IGHV4-34 and SLE clinical features.

## Methods

### Patient characteristics and assessment of disease activity

The Immune Cell Gene Expression Atlas from the University of Tokyo (ImmuNexUT), which is composed of 28 immune cell subsets including B cells from 10 categories of immune-mediated diseases, was analysed in this study [10]. The SLE patients who provided the samples in this database were recruited from the following three hospitals: Department of Allergy and Rheumatology at the University of Tokyo Hospital, Division of Rheumatic Diseases at the National Center for Global Health and Medicine, and the Immuno-Rheumatology Center at St Luke’s International Hospital. This study received approval from the Ethics Committee of the University of Tokyo (approval number: G10095), and all participants provided written informed consent.

All patients met the 1997 revised version of the ACR SLE criteria [11], and their disease activities were assessed using the SLEDAI 2000 (SLEDAI-2K) [12]. Patients administered CYC or rituximab within the past 1 year or taking ≥21 mg/day prednisolone were excluded from this analysis. Assessment of a lupus low disease activity state (LLDAS) was performed based on previously described criteria [2].

In this study, the patients were categorized as having decreased complement levels when the C3 or C4 level was below the institutional reference value. SLE flares were assessed based on the Safety of Estrogens in Lupus Erythematosus National Assessment (SELENA)-SLEDAI Flare Index [13]. Patients who experienced a ‘mild’ or more severe flare were classified into the flare group.

### Flow cytometry and gating strategy

Peripheral blood mononuclear cells were isolated from patients’ peripheral blood using Ficoll–Paque (GE Healthcare, Chicago, IL, USA). Ammonium-Chloride-Potassium (ACK) lysing buffer (ThermoFisher, Waltham, MA, USA) was used to eliminate erythrocytes, and Fc Receptor Binding Inhibitor Polyclonal Antibody (eBioscience, San Diego, CA, USA) was used in every experiment. The BD FACSAria Fusion flow cytometer (BD Biosciences, San Jose, CA, USA) was used for cell sorting.

In this study, our ImmuNexUT database was utilized for analysis. Therefore, the antibodies for flow-cytometry and gating strategy followed the same methods as previously described [10]. Briefly, for B cell analysis, first CD19^+^ peripheral blood mononuclear cells were sorted and divided into five subsets: naïve B cells (CD19^+^ IgD^+^ CD27^−^), unswitched memory (USM) B cells (CD19^+^ IgD^+^ CD27^+^), switched memory B cells (CD19^+^ IgD^−^ CD27^+^), double-negative B cells (CD19^+^ IgD^−^ CD27^−^) and plasmablasts (CD19^+^ IgD^−^ CD27^++^ CD38^+^).

### Acquisition of IGHV4-34 sequences from RNA-seq data

To analyse BCR, MiXCR software (version 3.0.13) was used, which enables the identification of the sequences of complementarity-determining regions (CDR) and framework regions from bulk RNA-sequencing (RNA-seq) data. BCR sequences, including IGHV4-34, were detected from RNA-seq FASTQ files [14]. Clonotypes were determined according to the CDR3 sequence. Usage of IGHV4-34 was defined by the following value: the number of clonotypes using IGHV4-34 gene divided by the total number of clonotypes in that sample. Receiver operating characteristic (ROC) curve analysis was used to formulate the threshold of IGHV4-34 usage in USM B cells which distinguished patients with low complement levels from the other patients.

### Statistical analysis

All numerical data are presented as medians with interquartile ranges (IQR) and categorical data as percentages, unless otherwise stated. Continuous variables were assessed using the Mann–Whitney non-parametric test and categorical variables using Fisher’s exact test. Correlation analysis was performed using Spearman’s rank correlation analysis to calculate statistical significance. Multiple comparisons were conducted using the Bonferroni correction. Statistical analyses were performed using R software (v. 4.2.1). Multiple linear regression was performed assuming a Gaussian distribution.

## Results

### IGHV4-34 usage in USM B cells reflects disease activity

**Figure 1.**
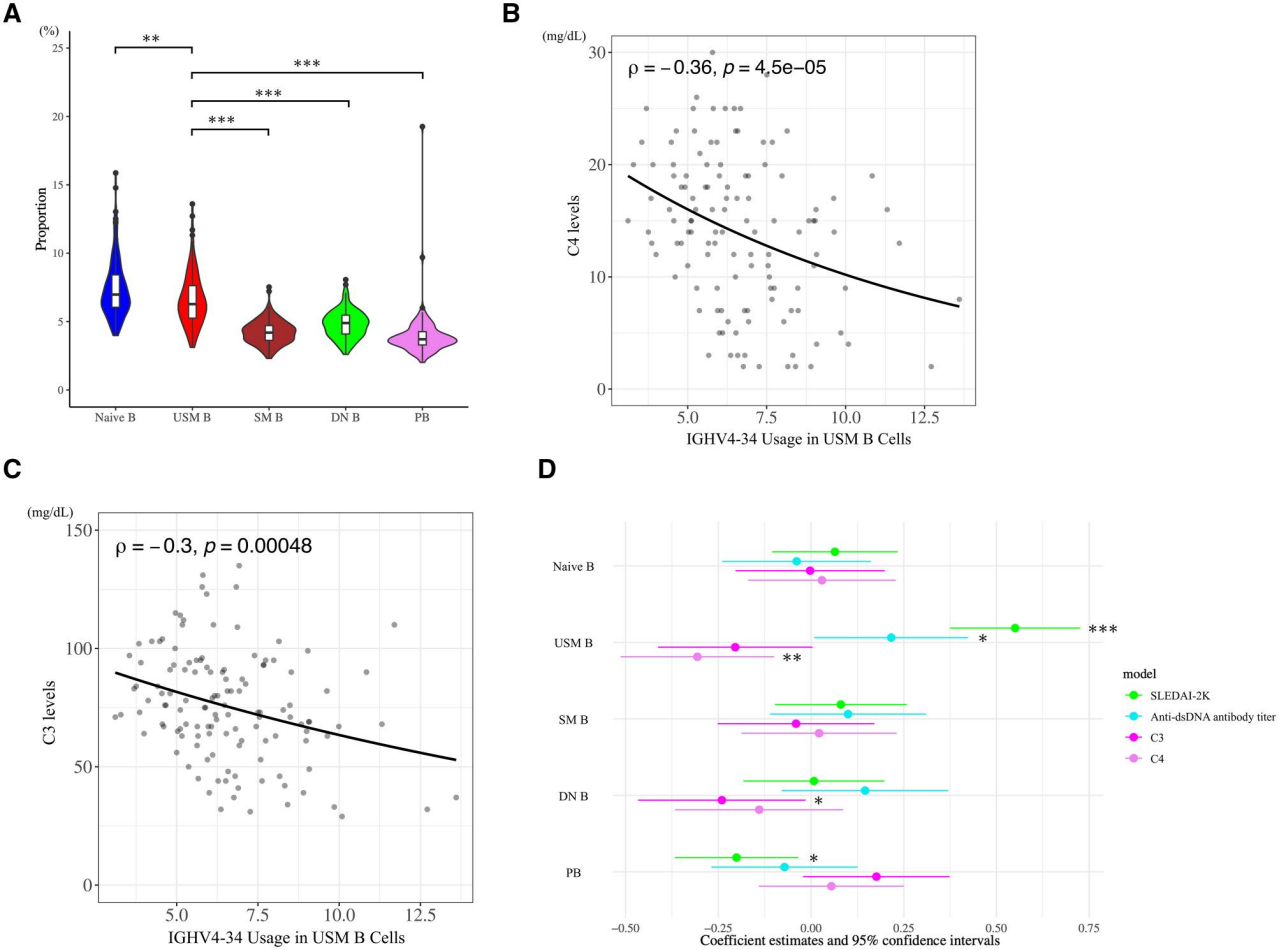
IGHV4-34 usage in USM B cells is associated with disease activity and decreased complement levels. (**A**) Violin plot depicting IGHV4-34 usage in each B-cell subset. Statistical significance was defined as *P < 0.05, **P < 0.01, ***P < 0.001 (adjusted *P*-values). Multiple comparisons were performed using the Bonferroni correction. Selected significance lines are shown. Complete results of significance analysis were shown in Supplementary Fig. S1, available at *Rheumatology* online. (**B**) Scatter plot showing the correlation between the C4 level and IGHV4-34 usage in USM B cells. The black line represents the regression line. The correlation coefficients and *P*-values were determined using Spearman’s rank correlation analysis. (**C**) Scatter plot showing the correlation between the C3 level and IGHV4-34 usage in USM B cells. The black line represents the regression line. The correlation coefficients and *P*-values were determined using Spearman’s rank correlation analysis. (**D**) Forest plot showing the estimated correlation between IGHV4-34 usage in each cell subset and clinical data. After data scaling, the CI was calculated by multiple linear regression analysis. Points represent the median values, and lines span the 95% CIs. *P < 0.05, **P < 0.01, ***P < 0.001. IGHV: immunoglobulin heavy-chain variable region; USM: unswitched memory; SM: switched memory; DN: double negative; PB: plasmablast

A total of 129 SLE patients were recruited, and the IGHV4-34 usage in each cell subset of the patients was analysed. Naïve B and USM B cells had higher IGHV4-34 usages compared with the other cell subsets, and IGHV4-34 usage was lower in USM B (6.6%) than in naïve B cells (7.4%) (Fig. 1A, Supplementary Fig. S1, available at *Rheumatology* online).

Correlation analysis revealed that IGHV4-34 usage in USM B cells was strongly correlated with the C4 level negatively (*P*-value <0.001), and it was the same with C3 (*P*-value <0.001) (Fig. 1B and C). Furthermore, USM B cells showed an especially significant association with the decreased C4 level, increased anti-dsDNA antibody titre and increased SLEDAI-2K score (Fig. 1D).

### IGHV4-34 usage in USM B cells could be a biomarker for stratification of SLE patients

**Figure 2.**
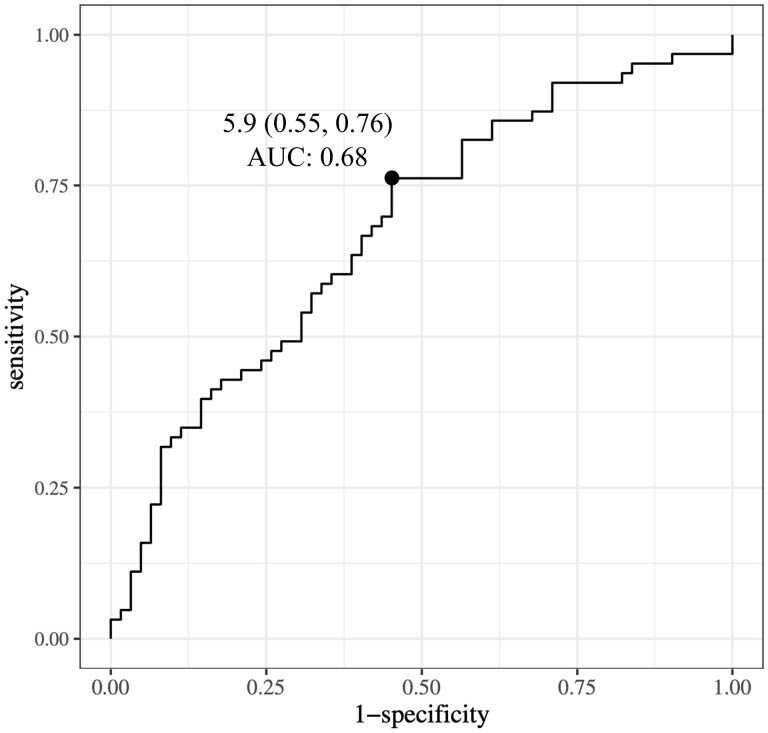
ROC curve demonstrating the ability of decreased complement levels to predict IGHV4-34 usage in USM B cells. Point represents the closest point to the top left corner. ROC: receiver operating characteristic; AUC: area under the curve; IGHV: immunoglobulin heavy-chain variable region; USM: unswitched memory

Next, we attempted to stratify SLE patients by IGHV4-34 usage. As described, IGHV4-34 usage in USM B cells was most correlated with decreased complement levels, so the cut-off for patient stratification by IGHV4-34 usage was evaluated according to the presence or absence of decreased complement levels. ROC curve analysis revealed that cut-off value of 5.9% was the most suitable to distinguish patients with low complement levels from the others (76.2% sensitivity, 54.8% specificity, 0.677 area under the curve) (Fig. 2). We used this threshold value to divide the SLE patients into two groups: high *vs* low IGHV4-34 usage in USM B cells.

### Comparison of IGHV4-34 usage between high and low IGHV4-34 usage patients

The clinical features and differences between the two SLE patient groups (high *vs* low IGHV4-34 usage in USM B cells) are shown in Tables 1 and 2. Arthritis, haematuria, proteinuria, rash and fever were more frequently observed in the high IGHV4-34 usage group. Regarding the laboratory findings, the high IGHV4-34 usage group had a higher ESRs, IgG and anti-dsDNA antibody titres, along with lower albumin and haemoglobin levels. Autoantibody titres also differed between the groups, with a greater rate of anti-Sm antibody positivity in the high IGHV4-34 usage group [high usage patients: 23 (30.3%) *vs* low usage patients: 4 (8.3%), *P-*value = 0.019]. There was no significant difference in the treatments used between the two groups.

**Table 1.** Clinical features of the high and low IGHV4-34 usage groups

	High IGHV4-34 usage in USM B cells (*n *= 80)	Low IGHV4-34 usage in USM B cells (*n *= 49)	*P*-value
Age, years, median (IQR)	42.0 (32.3, 52.3)	49.0 (42.0, 56.0)	0.041*
Male sex, *n* (%)	8 (10.0)	2 (4.08)	0.95
Disease duration, years, median (IQR)	11.0 (3.00, 17.0)	14.0 (5.00, 23.0)	0.087
Past history			
Lupus nephritis, *n* (%)	50 (62.5)	28 (57.1)	>0.99
Interstitial lung disease, *n* (%)	4 (5.00)	7 (14.3)	0.20
Laboratory data, median (IQR)			
AST (U/l)	20.0 (16.0, 25.0)	19.0 (16.0, 24.0)	>0.99
ALT (U/l)	15.0 (10.8, 21.0)	15.0 (11.0, 20.0)	>0.99
Cre (mg/dl)	0.67 (0.60, 0.78)	0.68 (0.63, 0.82)	>0.99
CRP (mg/dl)	0.085 (0.028, 0.45)	0.05 (0.030, 0.12)	>0.99
ESR (mm)	27.0 (14.0, 44.0)	13.0 (6.00, 21.0)	<0.0001***
Albumin (g/dl)	3.8 (3.3, 4.1)	4.0 (3.8, 4.3)	0.041*
IgG (mg/dl)	1636 (1219, 2040)	1239 (1071, 1480)	<0.01**
C3 (mg/dl)	70 (53, 88)	81 (68, 96)	0.026*
C4 (mg/dl)	12 (7.0, 17) (*n *= 78)	17 (14, 21) (*n *= 48)	<0.01**
Anti-dsDNA antibody titre (IU/ml)	14.1 (2.43, 60.6)	3.2 (0.80, 13.7)	<0.01**
UPCR (g/g Cre)	0.15 (0.060, 0.37)	0.10 (0.060, 0.27)	>0.99
WBC count (10^3^ cells/mm^3^)	4.9 (3.8, 6.6)	5.8 (4.5, 8.1)	0.18
Neutrophil count (10^3^ cells/mm^3^)	3.4 (2.4, 4.8)	4.8 (3.0, 5.8)	0.36
Lymphocyte count (10^3^ cells/mm^3^)	1.0 (0.67, 1.4)	1.1 (0.73, 1.4)	>0.99
Platelet count (10^9^ cells/l)	21.7 (17.1, 26.7)	22.8 (19.4, 25.3)	>0.99
Haemoglobin (g/dl)	11.8 (10.3, 12.9)	12.8 (11.9, 13.6)	0.019*
Rate of autoantibody positivity, *n* (%)			
ANA	80 (100)	49 (100)	>0.99
Anti-Sm antibody	23 (30.3)	4 (8.3)	0.019*
Anti-RNP antibody	35 (46.1)	12 (26.1)	0.18
Anti-SS-A antibody	63 (78.7)	29 (60.4)	0.21
Lupus anticoagulant	32 (40.0)	15 (31.9)	>0.99
Treatments			
Prednisolone, mg, median (IQR)	5.0 (2.4, 7.1)	5.0 (4.5, 5.4)	>0.99
Tacrolimus, *n* (%)	15 (18.8)	11 (22.4)	>0.99
Ciclosporin, *n* (%)	8 (10.0)	4 (8.16)	>0.99
MMF, *n* (%)	17 (21.3)	11 (22.4)	>0.99
AZA, *n* (%)	6 (7.5)	5 (10.2)	>0.99
MTZ, *n* (%)	1 (1.3)	1 (2.04)	>0.99
HCQ, *n* (%)	34 (42.5)	24 (49.0)	>0.99
Disease activity			
PGA, median (IQR)	1.1 (0.6, 1.8)	0.30 (0.30, 0.90)	<0.0001***
SLEDAI-2K, median (IQR)	6.0 (3.75, 12.0)	2.0 (0.00, 4.00)	<0.0001***
Achievement of LLDAS, *n* (%)	28 (35.0)	34 (69.4)	<0.001***

Data are shown as *n* (%) or median (IQR). Statistical significance was defined as

* *P* < 0.05,

** *P* < 0.01 or

*** *P* < 0.001.

Multiple comparisons were performed using the Bonferroni correction. IGHV: immunoglobulin heavy-chain variable region; IQR: interquartile range; USM: unswitched memory; AST: aspartate aminotransferase; ALT: alanine aminotransferase; UPCR: urine protein/creatinine ratio; WBC: white blood cell; PGA: physician global assessment; SLEDAI-2K: SLEDAI 2000; LLDAS: lupus low disease activity state.

**Table 2.** SLEDAI-2K manifestation of the high and low IGHV4-34 usage in USM B groups

	High IGHV4-34 usage in USM B cells (*n *= 80)	Low IGHV4-34 usage in USM B cells (*n *= 49)	*P*-value
Seizure	0 (0.00)	0 (0.00)	>0.99
Psychosis	0 (0.00)	0 (0.00)	>0.99
Organic brain syndrome	1 (1.25)	0 (0.00)	>0.99
Visual disturbance	4 (5.00)	0 (0.00)	0.30
Cranial nerve disorder	1 (1.25)	0 (0.00)	>0.99
Lupus headache	2 (2.50)	0 (0.00)	0.53
CVA	0 (0.00)	0 (0.00)	>0.99
Vasculitis	0 (0.00)	0 (0.00)	>0.99
Arthritis	22 (27.5)	4 (8.16)	0.012*
Myositis	1 (1.25)	0 (0.00)	>0.99
Urinary casts	12 (15.0)	0 (0.00)	<0.01**
Haematuria	17 (21.3)	1 (2.04)	<0.01**
Proteinuria	17 (21.3)	4 (8.16)	0.083
Pyuria	5 (6.25)	0 (0.00)	0.16
Rash	23 (28.8)	5 (10.2)	0.015*
Alopecia	14 (17.5)	6 (12.2)	0.46
Mucosal ulcers	6 (7.50)	1 (2.04)	0.25
Pleurisy	4 (5.00)	0 (0.00)	0.30
Pericarditis	2 (2.50)	0 (0.00)	0.53
Low complement	49 (61.3)	15 (30.1)	<0.01**
Increased DNA binding	39 (48.8)	11 (22.4)	<0.01**
Fever	15 (18.8)	2 (4.08)	0.017*
Thrombocytopenia	6 (7.50)	1 (2.04)	>0.99
Leukopenia	8 (10.0)	3 (6.12)	0.53

Data are shown as *n* (%). Statistical significance was defined as

* *P* < 0.05 or

** *P* < 0.01.

SLEDAI-2K: SLEDAI 2000; IGHV: immunoglobulin heavy-chain variable region; USM: unswitched memory; CVA: cerebrovascular accident.

Strikingly, the clinical features reflecting disease activity showed a significant difference between the groups. The SLEDAI-2K score [high usage patients: 6.0 (interquartile range 3.75, 12.0) *vs* low usage patients: 2.0 (0.00, 4.00), *P*-value <0.0001], physician global assessment score [high usage patients: 1.1 (0.6, 1.8) *vs* low usage patients: 0.30 (0.30, 0.90), *P*-value <0.0001] and rate of LLDAS achievement [high usage patients: 28 (35.0%) *vs* low usage patients: 34 (69.4%), *P*-value <0.001] all showed worse values in the high compared with the low IGHV4-34 usage group (Table 1).

**Figure 3.**
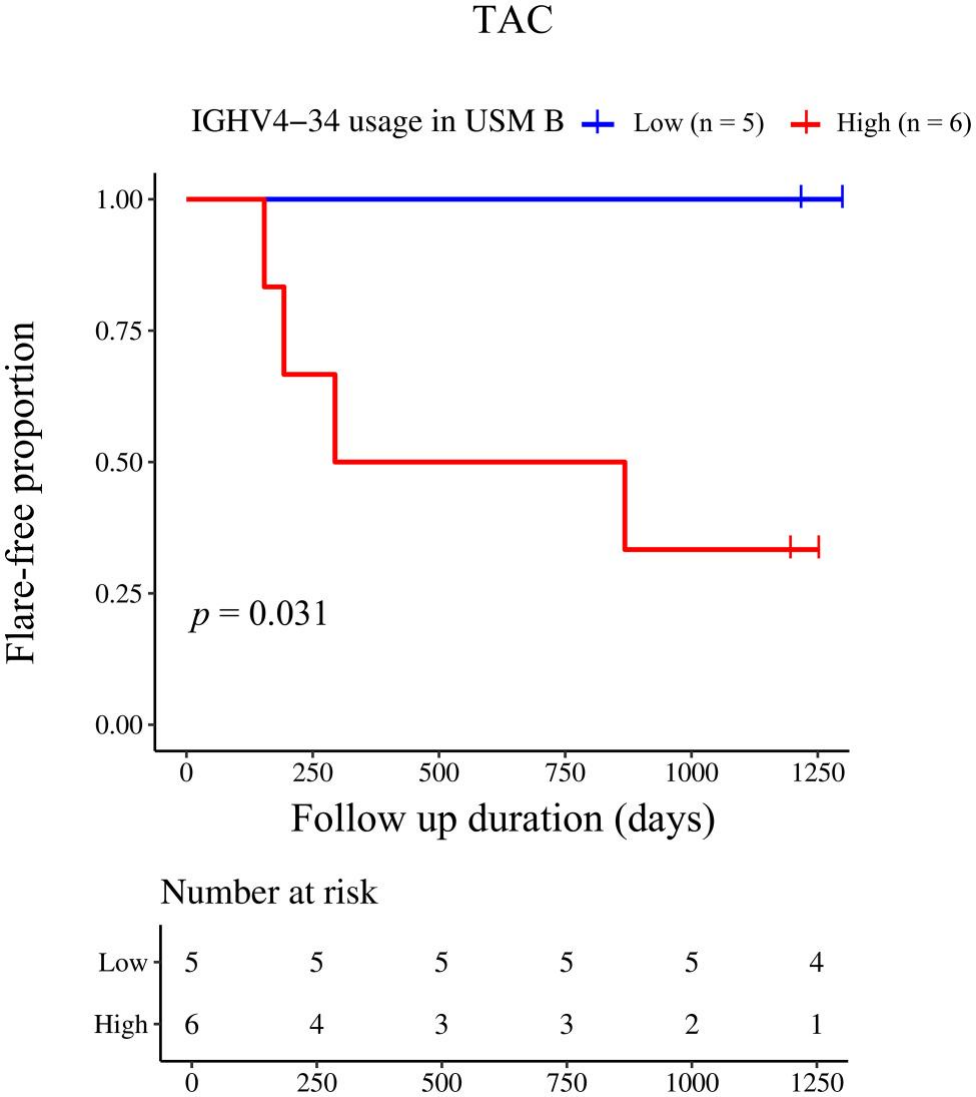
Kaplan–Meier curves comparing flare-free survival between the high and low IGHV4-34 groups among the patients who met the definition for LLDAS and were treated with tacrolimus. SLE flares were assessed based on the SELENA-SLEDAI Flare Index. The blue and red lines represent IGHV4-34 usage in the low and high IGHV4-34 usage groups, respectively. Statistical analysis was performed using the log-rank test. IGHV: immunoglobulin heavy-chain variable region; LLDAS: lupus low disease activity state; SELENA: Safety of Estrogens in Lupus Erythematosus National Assessment

Furthermore, we investigated the relationship between IGHV4-34 usage in USM B cells and the risk of an SLE flare. Because immunosuppressive drugs used for maintenance treatment can affect the SLE flare risk [15, 16], we evaluated the rate of flare-free survival for each immunosuppressant. Among the patients treated with tacrolimus, higher IGHV4-34 usage in USM B cells was associated with a significantly higher flare rate (*n *= 11, Fig. 3), suggesting that IGHV4-34 usage in USM B cells is a potential biomarker of SLE flares. On the other hand, among the patients treated with MMF or HCQ, there was no significant difference in the flare rate between the high and low IGHV4-34 usage in USM B cells groups (Supplementary Fig. S2A and B, available at *Rheumatology* online).

## Discussion

This study demonstrated that IGHV4-34 usage in USM B cells was a potential biomarker to stratify SLE patients. Patients with high IGHV4-34 usage in USM B cells showed severe disease activity associated with arthritis, haematuria, proteinuria, rash and fever.

The abnormal quality and quantity of USM B cells in SLE and the associations of these abnormalities with autoantibodies or disease activity have been reported previously. The number of USM B cells is decreased in SLE patients compared with healthy controls, and it is positively correlated with the white blood cell count. In addition, the number of USM B cells is correlated with serum complement levels and inversely with the SLEDAI score and anti-dsDNA antibody titre [17]. Our study reinforces the findings of previous studies and provides insight into the mechanism of USM B cell contribution to SLE.

Furthermore, IGHV4-34 is one of the most autoreactive sequences, and its association with SLE has been reported. IGHV gene usage showed changes in autoimmune diseases including serum IGHV4-34 titres and IGHV4-34^+^ B cells expanded in SLE [18]. Our data showed the potential of IGHV4-34 usage in USM B cells as a biomarker to stratify SLE patients. Achievement of LLDAS may reduce overall organ damage, which is one of the goals of SLE treatment [2], and the importance of proper patient stratification is underscored. However, at present, a biomarker that can distinguish LLDAS patients from other SLE patients has not been identified. Our study highlighted the potential of IGHV4-34 usage in USM B cells as such a biomarker. We found no difference in the treatments used between the high and low IGHV4-34 usage groups. However, disease activity showed a drastic contrast between the groups, suggesting an association between high IGHV4-34 usage in USM B cells and refractoriness to treatment. Considering this finding, the existing treatments have an inadequate effect on IGHV4-34, and IGHV4-34 could be a new target for therapy.

The role of USM B cells in antibody production has not been fully elucidated. However, it is suggested that USM B cells are products of the germinal centre-independent pathway to provide protection against foreign invaders during the early phase [19]. In addition, it is possible that membrane-bound BCR itself can activate complement proteins including C3 or C4 [20]. These findings may explain why IGHV4-34 usage in USM B cells is strongly associated with complement levels.

Although this study had a limited number of patients, SLE recurrence under tacrolimus treatment happened more frequently in patients with high IGHV4-34 usage in USM B cells. Tacrolimus is a calcineurin inhibitor that affects T cells by inhibiting calcineurin, MAPK signalling and TGFβ signalling; its effects on B cells are limited [21, 22]. Among patients with high IGHV4-34 usage in USM B cells, there are unknown pathological signalling pathways that cannot be controlled by tacrolimus, potentially leading to relapse. Therefore, tacrolimus potency as maintenance therapy in SLE patients with high IGHV4-34 usage in USM B cells may be poor, so IGHV4-34 usage in USM B cells could serve as a biomarker for patient stratification.

This study has some limitations. First, recent studies revealed cell subsets that contribute to autoantibody production, such as DN2 cells [23], and more detailed research on other B-cell subsets is needed. Next, BCR diversity is influenced by not only VDJ gene usage but also SHM and class switching. Several autoreactive motifs for IGHV4-34 (e.g. AVY and NHS motifs) have been reported [9], and SHM can disrupt IGHV4-34 autoreactivity. These factors could be important covariates. The efficacy of immunosuppressants, including tacrolimus, on SLE flares was assessed in relatively few patients, so validation in larger samples is expected.

Despite these limitations, this study is the first to demonstrate the ability to stratify SLE patients by VDJ gene usage, and the findings provide new insight into treatment strategy for SLE.

## Supplementary Material

keaf386_Supplementary_Data

## Data Availability

The data that support the findings of this study are available from the corresponding author, K.F., upon reasonable request.
