# Supplementary material for: Stratification of systemic lupus erythematosus with IGHV4-34 in unswitched memory B cells
Source: Rheumatology (Oxford). 2025 Jul 12;64(12):6099–105. doi: 10.1093/rheumatology/keaf386 (PMC12671857; doi:10.1093/rheumatology/keaf386)
Supplement: keaf386_Supplementary_Data [file keaf386_supplementary_data.docx]

**Supplementary Figure S1:** Violin plot depicting IGHV4-34 usage in each B cell subset.

**
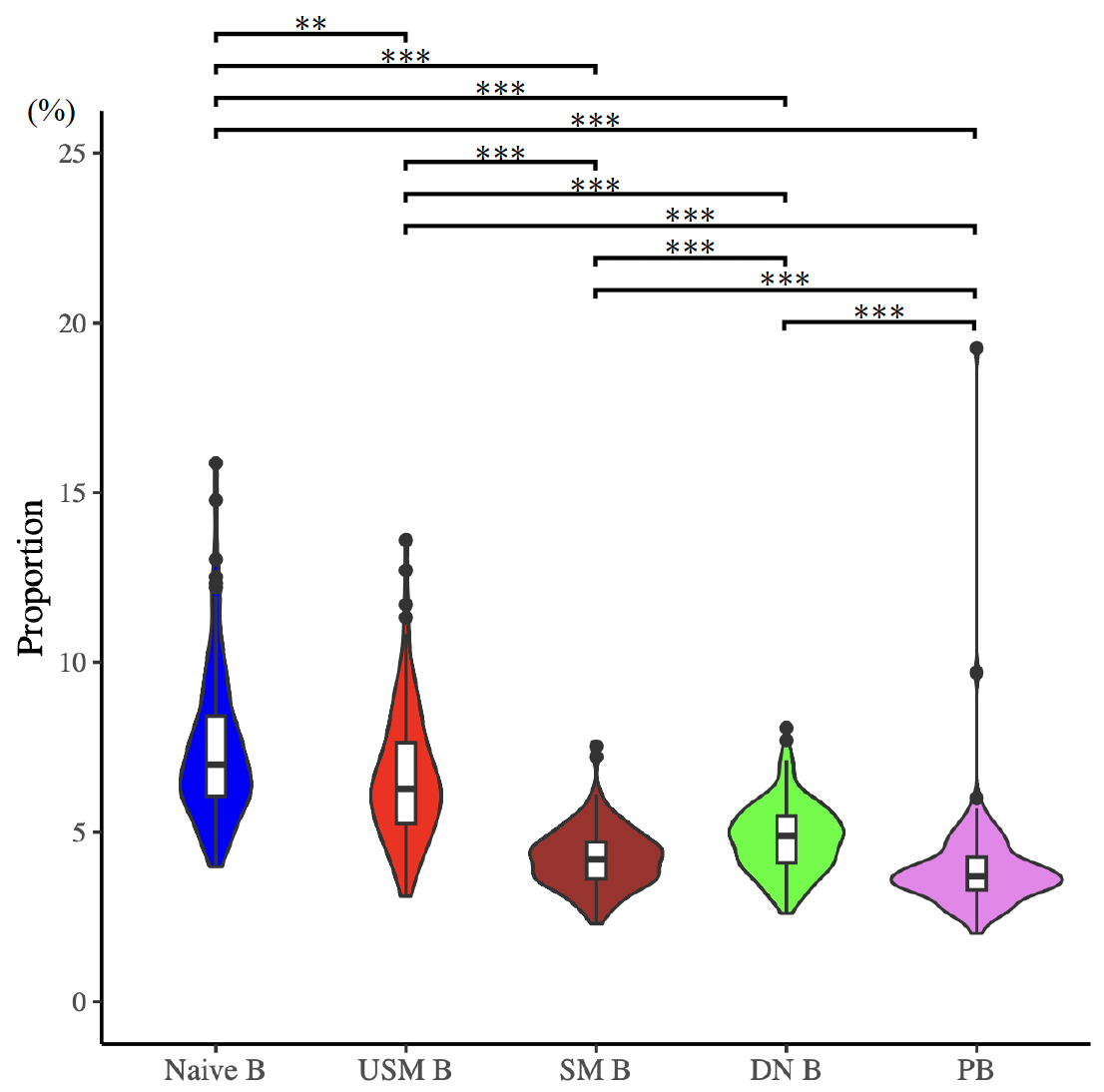
**

Statistical significance was defined as **p* < 0.05, ***p* < 0.01, ****p* < 0.001 (adjusted *p*-values). Multiple comparisons were performed using the Bonferroni correction.

ALT TEXT: The graph is a violin plot displaying IGHV4-34 usage in each B cell subset, and naive B and USM B cells show higher IGHV4-34 usage compared with the other cell subsets. In contrast, PB exhibits lower IGHV4-34 usage.

**Supplementary Figure S2:** Kaplan–Meier curves comparing flare-free survival between the low and high IGHV4-34 usage groups among the patients treated with mycophenolate mofetil or hydroxychloroquine.

1. Mycophenolate mofetil.


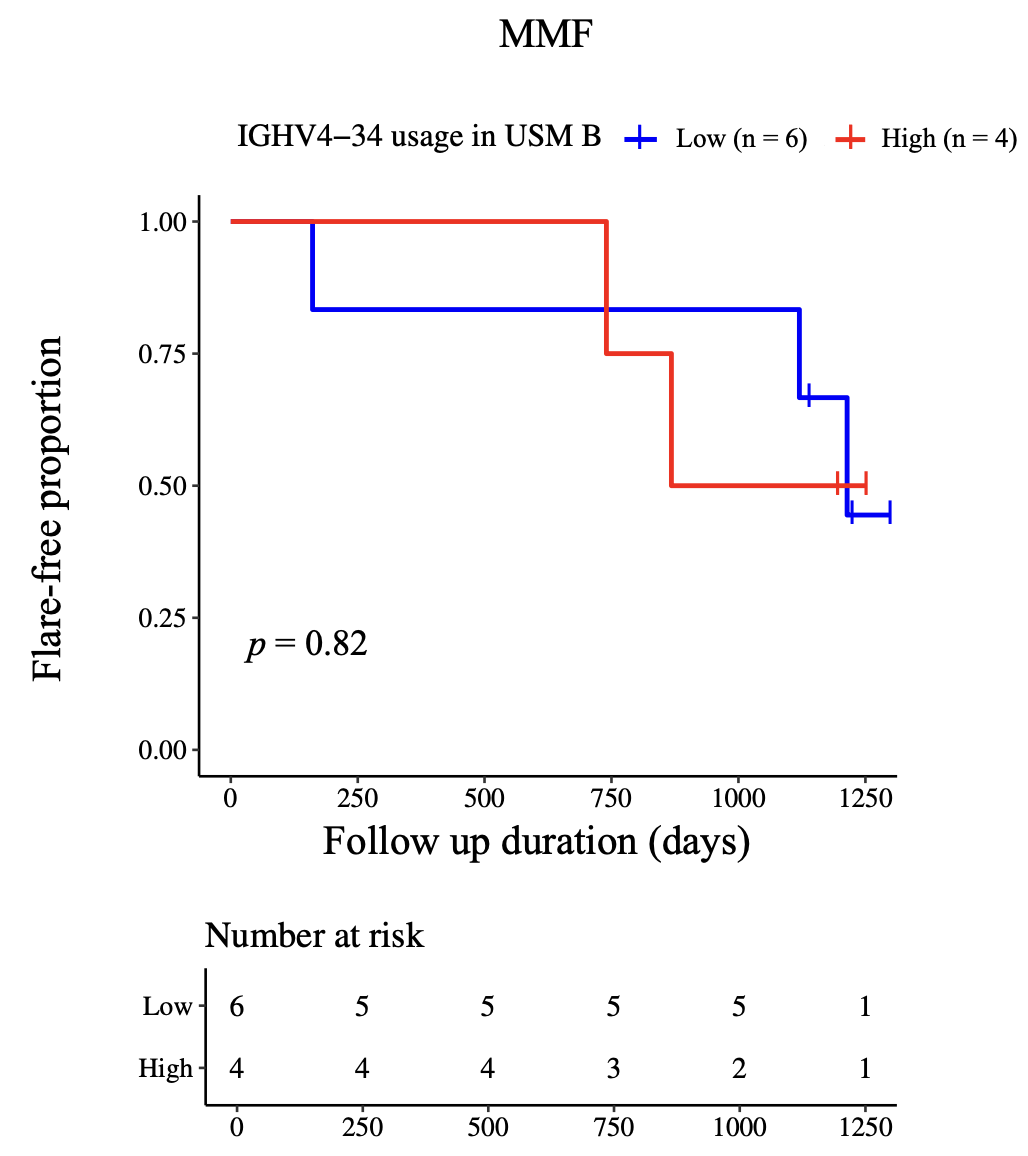


1. Hydroxychloroquine.


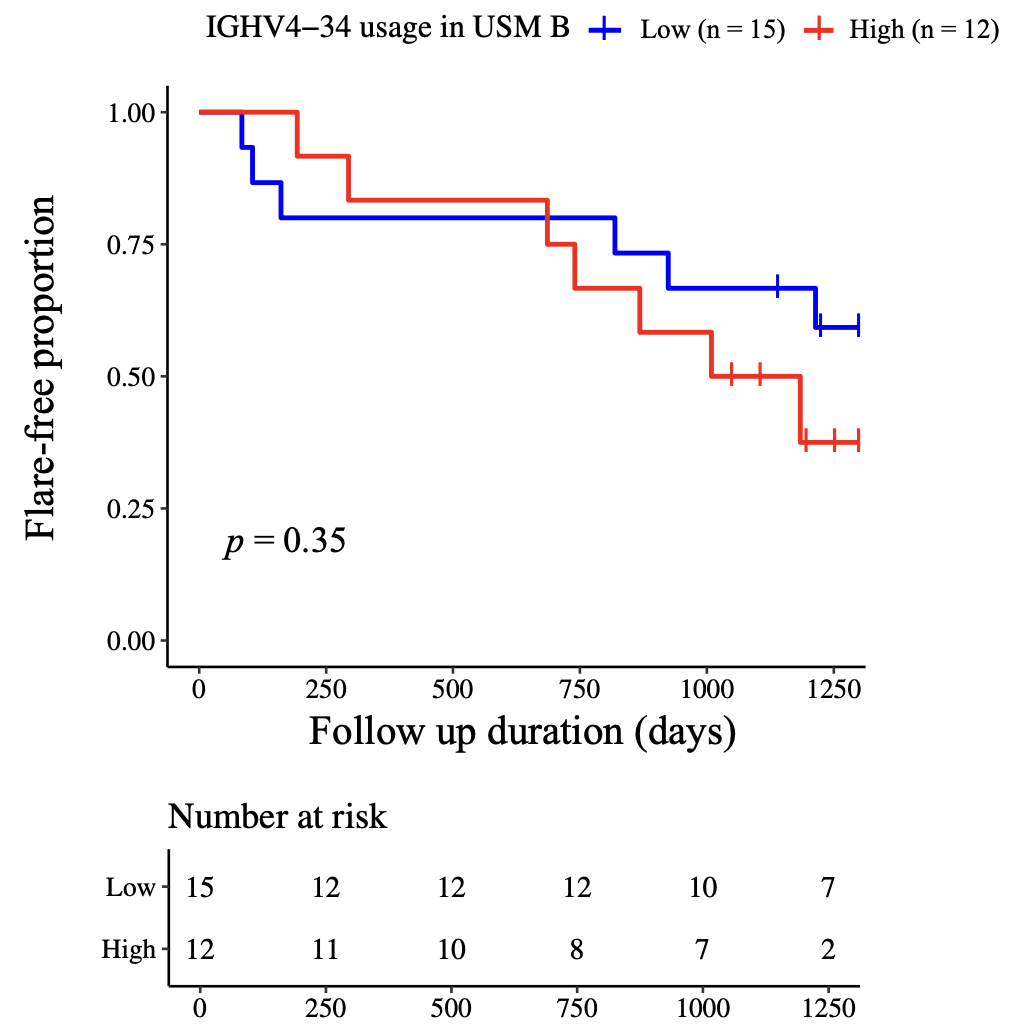


SLE flares were assessed based on the Safety of Estrogens in Lupus Erythematosus National Assessment (SELENA)-SLEDAI Flare Index. Blue and red lines represent IGHV4-34 usage in the low and high IGHV4-34 usage groups. Statistical analysis was performed using the log-rank test.

ALT TEXT: Kaplan–Meier curves comparing flare-free survival under mycophenolate mofetil or hydroxychloroquine treatment between patients with high and low IGHV4-34 usage in USM B cells. There is no difference in flare rate between patients with high and low IGHV4-34 usage in USM B cells.
